# Novel Hybrid Gel–Fiber Membranes as Carriers for Lipase Catalysis Based on Electrospinning and Gelation Technology

**DOI:** 10.3390/gels10010074

**Published:** 2024-01-18

**Authors:** Shumiao Lin, Qianqian Zhang, Ziheng Wang, Jinlong Li

**Affiliations:** 1 Beijing Engineering and Technology Research Center of Food Additives, Beijing Technology and Business University, Beijing 100048, China; linshumiao999@163.com (S.L.); 18852862233@163.com (Q.Z.); 2 Key Laboratory of Green Manufacturing and Biosynthesis of Food Bioactive Substances, China General Chamber of Commerce, Beijing 100048, China; henghengheng2021@163.com; 3 School of Food and Health, Beijing Technology and Business University, Beijing 100048, China

**Keywords:** hybrid gel–fiber membranes, interfacial catalysis, lipases, electrospinning, gelation

## Abstract

An excellent oil–water interface is one of the prerequisites for effective lipase catalysis. Therefore, this study aimed to improve lipase activity in terms of catalytic interface optimization. A novel approach for constructing oil–water interfaces was proposed. The structural similarity and the hydrophilic differences between polyvinyl pyrrolidone gel–fiber membranes (GFMs) and poly(lauryl methacrylate) (PLMA) organogel inspired us to hybridize the two to form PVP/PLMA hybrid gel–fiber membranes (HGFMs) based on electrospinning and gelation. The prepared PVP/PLMA-HGFMs were capable of being adopted as novel carriers for lipase catalysis due to their ability to swell both in the aqueous phase (swelling ratio = 187.5%) and the organic phase (swelling ratio = 40.5%). Additionally, Confocal laser scanning microscopy (CLSM) results showed that abundant network pores inside the carriers enabled numerous effective microscopic oil–water interfaces. The catalytic activity of *Burkholderia cepacia* lipase (BCL) in PVP/PLMA-HGFMs ranged between 1.21 and 8.70 times that of the control (“oil-up/water-down” system) under different experimental conditions. Meanwhile, PVP/PLMA-HGFMs increased lipase activity by about eight times at −20 °C and had good application characteristics at extreme pH conditions.

## 1. Introduction

Lipase, a triacylglycerol hydrolase, has been increasingly studied and applied in biochemistry, pharmaceutical development, food engineering, and biosensors [1,2,3,4,5,6,7]. Unlike other enzymes, most lipases are compounds that are incompatible or only minimally compatible in terms of polarity with their substrates, and the catalytic reaction takes place at the interfacial junction of the two media, which is known as the interfacial catalytic mechanism of lipases [8,9]. From the molecular structure point of view, the essence of this special catalytic mechanism lies in the amphipathic polypeptide chain (also known as the “lid” structure) covering the catalytic activity center of lipase, which consists of an external polar amino acid and an internal nonpolar amino acid [10,11,12,13]. Therefore, the active center of lipase is only able to function when it is at the interface of the two phases [14]. Overall, the interface is one of the important factors affecting the catalytic performance of lipases [15].

It is worth mentioning that the outreach of the influence of the oil–water interface on catalytic reactions from a broader perspective is not limited to biocatalysis. In the Beattie et al. report, changing the intensity of stirring could alter the interface area, thereby affecting the Diels–Alder reaction of CpH and dimethyl fumarate in the aqueous phase [16]. Although they did not report droplet size, the results are still instructive [17]. By investigating the correlation between stirring rate, the oil–water interfacial area, and rate constants, Manna and Kumar found that the enthalpy of a chemical reaction is insensitive to the hydrogen-bonding capacity of water molecules under homogeneous conditions, whereas the exothermic nature of the enthalpy decreases significantly with the reduction of the hydrogen bonding potential under nonhomogeneous conditions [17,18]. The system reported by Mellouli et al. could monitor the cycloaddition reaction of quadricyclane with diethyl azodicarboxylate (DEAD), which allowed the study of interfacial reactions on a macroscopic scale [19]. The oil–water interface was considered the main contributor, as the experimental results showed that the oil–water interface clearly accelerates the reaction rate to the same level observed in bulk solution without vigorous agitation [17,19]. Biphasic systems could also be used to enhance the cycloaddition of CO_2_ and the catalyst could be easily recovered at the end of the reaction [20].

As a result, the exploration of superior catalytic interfaces to enhance the catalytic activity of lipases has gradually become one of the focal points of research. The traditional macroscopic catalytic system of “oil-up/water-down” has the advantages of simple preparation and convenience, but its catalytic efficiency is not satisfactory. Emulsion systems, such as microemulsions and Pickering emulsions, have been studied more intensively because of their simple interface preparation ideas [21]. Stephanie et al. [22] stabilized *Candida antarctica* lipase B in a high internal phase emulsion and increased enzyme activity by 39%. However, while these systems improved the catalytic efficiency of lipases through “water-in-oil” (W/O) or “oil-in-water” (O/W) structures [6,23], they also suffered from shortcomings such as products that were not easily purified, complex preparation processes, and poor environmental stability [24,25]. In contrast, enzyme immobilization technology has more obvious technical advantages than the emulsion method [26]. Whether specific or nonspecific binding, lipases were able to be reused with advanced catalytic efficiency and improved stability depending on the carrier [27]. However, it cannot be denied that more studies have focused on the application properties of lipases, but few reports have taken the catalytic interface as an entry point.

The catalytic efficiency of emulsion systems is higher than that of “oil-up/water-down” systems due to the creation of miniaturized catalytic interfaces and increased dimensions from 2D to 3D in the catalytic space within the W/O or O/W structure. On the other hand, however, the maximum catalytic space that can be utilized by a particular catalytic system (such as emulsion systems) is theoretically limited. Therefore, how to make “unlimited use” of the limited space is the underlying logic and fundamental contradiction that need to be focused on. In our previous research, the lipase catalytic system based on electrospinning membranes or gels increased the activity of lipase by approximately one to two times [28,29]. Both fiber membranes and gels had a fine and rich network structure at the microscopic level, which was the structural basis for the creation of catalytic interfaces. This structural similarity led us to wonder if the two could be hybridized to maximize the advantages of both.

**Figure 1:**
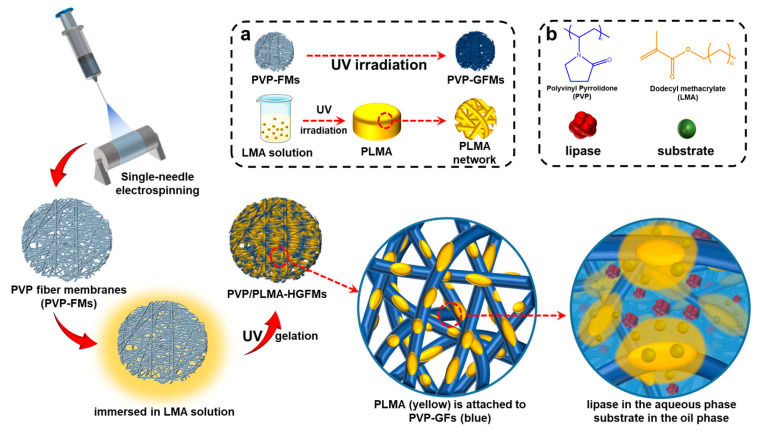
Illustration of PVP/PLMA-HGFMs as a carrier for lipase catalysis. Blue represents the aqueous phase and hydrophilic fibers, and yellow represents the organic phase and organogel. (**a**) When exposed to UV radiation, PVP-FMs will transform into PVP-GFMs, and LMA will transform into PLMA. (**b**) The substrate and lipase schematic diagrams and the raw material structural formula.

Thus, in the current study, we further expanded our research idea of constructing lipase-membrane reactors with efficient catalytic performance based on electrospinning and gelation technology and combined the two through the design idea in Figure 1. The organogel monomer LMA was permeated into the electrospun PVP fiber membranes (PVP-FMs, hydrogel precursor PVP as electrospinning solution), and then the PVP gel–fibers (PVP-GFs) and the PLMA were created by the UV radiation-induced gelation simultaneously, fabricating PVP/PLMA-HGFMs. This way, the PVP/PLMA-HGFMs would possess interlaced networks and a lot of pores, and the PVP-GFs and PLMA in the PVP/PLMA-HGFMs were able to swell both in the aqueous phase (contain lipases) and the organic phase (contain substrates). Consequently, the aqueous phase (containing lipases) could contact organic phase (containing substrates) within each of the internal micropores of PVP/PLMA-HGFMs and, thus, form effective catalytic interfaces. This is attributed to the fact that the pores were tiny and innumerable in the PVP/PLMA-HGFMs, and countless oil–water interfaces will be constructed for lipase catalysis.

Therefore, the combination of PVP-GFs and PLMA based on electrospinning may be a promising solution to optimize lipase catalysis. This work provides new perspectives on the idea of constructing catalytic interfaces for future research.

## 2. Results and Discussion

### 2.1. Gelation Mechanism of PVP/PLMA-HGFMs

**Figure 2:**
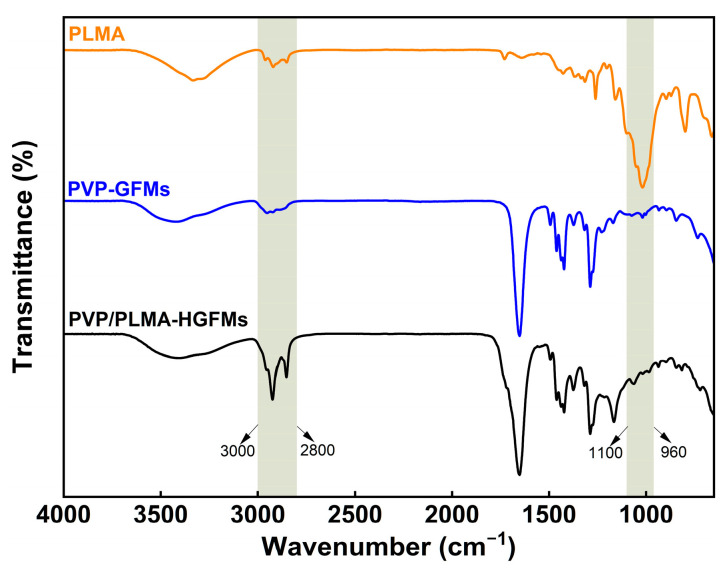
FTIR spectra of PVP-GFMs, PLMA organogel and PVP/PLMA-HGFMs.

In Figure 2, there were obvious absorption peaks at 3000–2800 cm^−1^ for PVP-GFMs, PLMA, and PVP/PLMA-HGFMs, which were mainly caused by the stretching vibration of saturated carbon (-CH_2_-) in all three. In addition, the transmittance of PVP/PLMA-HGFMs could be clearly observed to be relatively higher than that of PVP-GFMs and PLMA in this band. Such experimental results showed that the gelation process of PVP-FMs and LMA was adequate and thorough. It can be assumed that if the gelation was not sufficient, there would be a large amount of unsaturated C=C remaining and a reduced amount of saturated -CH_2_- in the respective free radical polymerization of PVP-FMs and LMA. This would lead to a lower intensity of the absorption peaks of the PVP/PLMA-HGFMs in the 3000–2800 cm^−1^ band. However, this was contrary to the experimental results. It should be noted that current FTIR results cannot directly confirm whether PVP-FMs and LMA successfully formed PVP-GFs and PLMA, respectively, or whether a block polymerization reaction occurred during the gelation process.

Compared to PLMA, the strong absorption peaks in the 1100–960 cm^−1^ band disappeared in PVP/PLMA-HGFMs. The absorption peak of the PLMA here was the result of the bending vibration of the PLMA side chains, which were saturated carbon chains consisting of 12 carbons. However, when PLMA and PVP-GFs were hybridized to form PVP/PLMA-HGFMs, PLMA was subjected to the mutually exclusive and hydrophobic interactions of PVP-GFs, which led to a limitation of the range of activity of the side chains and a weakening of the bending vibrations. Furthermore, it resulted in the disappearance of the absorption peaks here. Therefore, the above results implied that the hybridization process of PLMA and PVP-GFs was successful.

In addition, no new characteristic peaks appeared after hybridization, indicating that the PVP-FMs and LMA did not undergo new chemical reactions during gelation to produce additional functional groups in the free radical polymerization reaction.

### 2.2. Microstructural Features of PVP-GFMs, PLMA and PVP/PLMA-HGFMs

**Figure 3:**
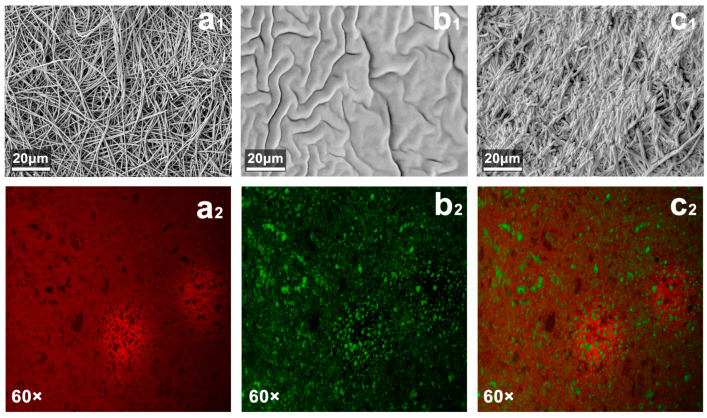
SEM images (**a_1_**–**c_1_**) and CLSM images (**a_2_**–**c_2_**) of different membranes. The SEM (**a_1_**) and CLSM images (**a_2_**) of PVP-GFMs, the SEM (**b_1_**) and CLSM images (**b_2_**) of PLMA organogel and the SEM (**c_1_**) and CLSM images (**c_2_**) of PVP/PLMA-HFGMs. The magnification of the SEM is 500×.

Under the SEM, the fiber diameter of PVP-GFMs was fine (>500 nm) and uniform (Figure 3a_1_). The cross-section of PLMA showed more folds and its surface roughness was high (Figure 3b_1_). This uneven structure helped the PLMA further absorb organic solvents. When PVP-FMs absorbed the LMA organogel precursor solution and further formed PVP/PLMA-HFGMs, they did not exhibit a well-defined morphology of the fiber network like the PVP-GFMs but only uniform and thicker-diameter gel–fibers (Figure 3c_1_). It is worth mentioning that comparing a_1_ with c_1_, the dimensions of the pores became smaller after the PVP-FMs were transformed into PVP/PLMA-HFGMs by hybridization, which indicated that the pores among the PVP-GFs were occupied partially by the cross-linked PLMA. This not only verified the successful cross-linking of the PLMA but also indicated the presence of a large amount of PLMA on the surface of the PVP-GFs in the PVP/PLMA-HFGMs.

**Figure 4:**
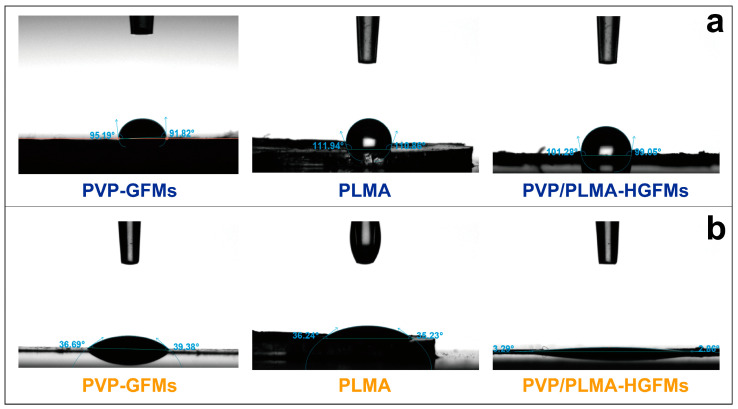
Water contact angle (**a**) and oil contact angle (**b**) of PVP-GFMs, PLMA and PVP/PLMA-HGFMs.

In the CLSM evaluation, PVP-GFMs and PLMA were able to be stained red and green by Nile Blue and Nile Red, respectively (Figure 3a_2_,b_2_). As shown in Figure 3c_2_, the red regions were spread throughout the whole image while the green regions were distributed in dots, which not only indicated that the PVP/PLMA-HGFMs were hybridized from PVP-GFs and PLMA organogel but also implied the hybridization was homogeneous on a microscopic scale. In addition, a large number of tiny green parts were found in the crevices of PVP/PLMA-HGFMs, which suggested PVP-GFs have swelling capacity for organic solvents (Figure 4b).

### 2.3. Capacity of Wetting and Swelling of PVP-GFMs, PLMA and PVP/PLMA-HGFMs

By comparing the size of the angle at the moment of contact, it is possible to understand the wettability of the material in the medium [30,31]. The larger the contact angle, the weaker the wettability [32].

The oil contact angles of PVP-GFMs, PLMA, and PVP/PLMA-HGFMs were all much smaller than their respective water contact angles (Figure 4a,b). This meant these three were more likely to wet oil than water. As shown in Figure 4a, the water contact angle of PVP/PLMA-HGFMs was intermediate between that of PLMA and PVP-GFMs. This was the result of the interaction of two mutually exclusive properties within PVP/PLMA-HGFMs: the hydrophobicity of the PLMA and the hydrophilicity of the PVP-GFs. Differently, the oil contact angle of PVP/PLMA-HGFMs was smaller than that of PLMA and PVP-GFMs (Figure 4b). PVP/PLMA-HGFMs had a lipophilic gel component compared to PVP-GFMs and had many microscopic fibers and pore structures compared to PLMA, so the two components of PVP/PLMA-HGFMs have a synergistic effect in the oil penetration process.

### 2.4. Swelling Ratio and Lipase-Holding Capacity of PVP/PLMA-HGFMs

**Figure 5:**
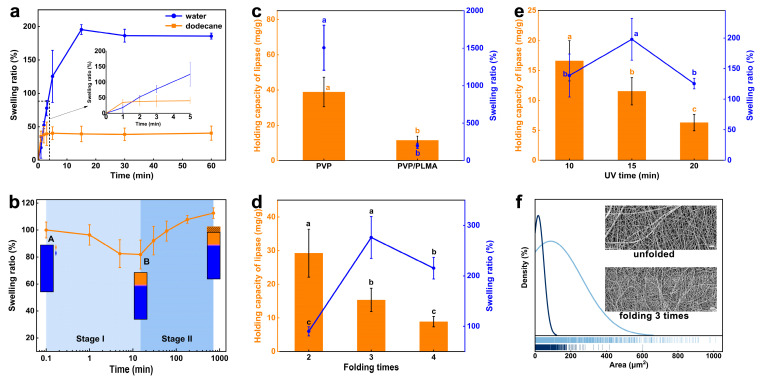
Swelling ratio and lipase-holding capacity of PVP/PLMA-HGFMs. (**a**) Water and oil swelling ratio of PVP/PLMA-HFGMs, respectively. (**b**) Swelling curves of PVP/PLMA-HGFMs in dodecane after saturated water swelling. Black rectangular box represented the internal space of PVP/PLMA-HGFMs at saturated swelled water, and blue and yellow represented water and oil, respectively. Effect of PLMA addition (**c**), different PVP-FMs folding times and (**d**) different UV irradiation time (**e**) on water swelling ratio and lipase-holding capacity. (**f**) Figure of the size and frequency distribution of pores after the formation of hybridized PVP/PLMA-HGFMs for different times of folding of PVP-FMs, respectively. Dark blue line represents folded three times and light blue line indicates unfolded. Compared to unfolded, the PVP/PLMA-HGFMs formed after folding three times had smaller pores, more concentrated frequency distribution and more uniform pore state. This was more conducive to the formation of microscopic oil–water interfaces. The scale of the SEM in the figure is 10 μm. Different letters in the same group indicate significant differences (*p* < 0.05).

Although the instantaneous oil-wetting capacity of PVP/PLMA-HGFMs was higher than the water-wetting capacity, the water swelling ratio of PVP/PLMA-HGFMs was approximately four times higher than the oil swelling ratio when PVP/PLMA-HGFMs reached the swelling equilibrium in the aqueous phase (187.5%) and organic phase (40.5%), respectively. This meant that PVP/PLMA-HGFMs had a high water swelling ratio and a high speed of oil swelling (Figure 5a).

Figure 5b simulated the change in swelling ratio when PVP/PLMA-HGFMs held lipases for catalysis. PVP/PLMA-HGFMs first swelled in the aqueous phase until saturation and were then permeated with dodecane. The change in swelling ratio went through two stages. In stage I, the swelling ratio first decreased slightly. We inferred that a small portion of the free water inside the PVP/PLMA-HGFMs was extruded by the organic phase. At the same time, this space was rapidly replaced by an equal volume of the organic phase, which in turn led to a decrease in the ratio of swelling because ρ_oil_ < ρ_water_. Point B was the lowest point of the curve, which meant the process of replacing water with oil had reached its end. Subsequently, the swelling ratio increased gradually at stage II. This was due to the fact that although the swelling of PVP/PLMA-HGFMs was saturated at point A, there still existed a part of the lipophilic space that was not filled by water. So, the organic phase entered this part of the space after the end of the first stage of replacement, leading to an increase in the swelling ratio.

**Figure 6:**
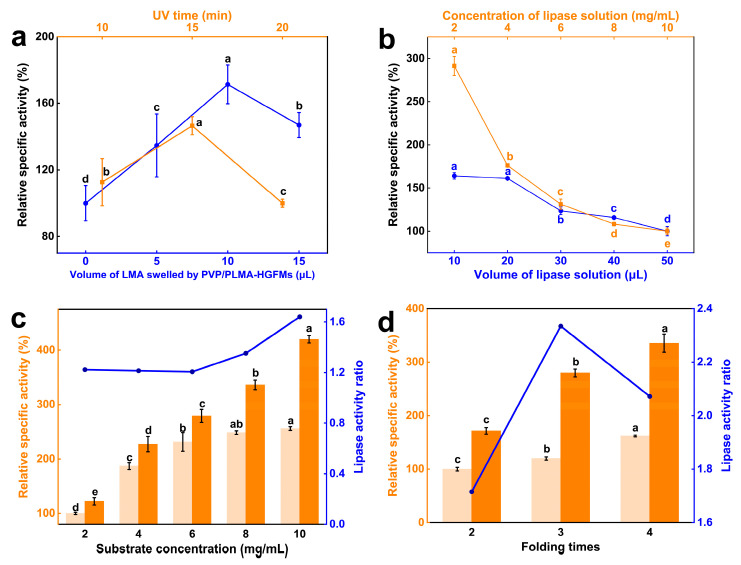
Catalytic properties of lipase in PVP/PLMA-HGFMs. Effect of LMA swelling and UV irradiation time (**a**), concentration (volume = 10 μL) and volume (concentration = 2 mg/mL) of lipase solution (**b**), substrate concentration (**c**) and folding times of PVP-FMs (**d**) on the relative specific viability of lipase. (**c**,**d**) The light yellow and orange in the bar graphs are for the control (“oil-up/water-down” system) and the experimental group (PVP/PLMA-HGFMs), respectively. Different letters in the same group indicate significant differences (*p* < 0.05).

Lipase enters the aqueous phase and disperses in a fibrous network space during carrier swelling, as it has a high degree of freedom and dispersion in the aqueous phase [6]. Furthermore, a high lipase-holding capacity requires a high water-holding capacity of the carrier [29]. Thus, the addition of hydrophobic PLMA organogel to the hydrophilic PVP-FMs not only resulted in a significant decrease in water-swelling capacity but also resulted in a significant decrease in the lipase-loading capacity of the PVP/PLMA-HGFMs (Figure 5c). It was worth mentioning that PVP/PLMA-HGFMs, which had lower water-swelling and lipase-holding capacity than PVP-GFMs, did not necessarily have lower lipase activity than PVP-GFMs. This will be discussed again in the next section (Figure 6a).

Since the mechanical strength of monolayer PVP-FMs was not sufficient to form PVP/PLMA-HGFMs, we increased the thickness and mechanical strength of PVP-FMs by folding them and explored the effect of this change on the water swelling ratio and lipase-holding capacity of PVP/PLMA-HGFMs. The lipase-holding capacity of PVP/PLMA-HGFMs decreased with increasing folding times (Figure 5d). This was because increasing the folding number of PVP-HGFMs led to a denser network, smaller pores (Figure 5f), and a smaller specific surface area. These factors led to an increase in the mass transfer resistance of PVP/PLMA-HGFMs and made it difficult for lipases to be distributed in the internal space. Thus, the efficiency of PVP/PLMA-HGFMs in holding lipases was reduced.

Unlike the above phenomenon, the water swelling ratio of PVP/PLMA-HGFMs showed a tendency to increase and then decrease and reached the optimal value at its folding number of three. This suggested that although there was some synergistic relationship between the water swelling ratio and the lipase-holding capacity (Figure 5c), this synergistic change was not immediately apparent. We believe that the reason for this was similar to the working mechanism of gel chromatography in terms of molecular dynamics. The mass transfer resistance from the increased folding number was negative for lipases and water molecules to enter the interior of PVP/PLMA-HGFMs. Specifically, mass transfer resistance impeded large molecules (lipases) more than small molecules (water molecules). This meant that lipases were more “sensitive” to mass transfer resistance than water molecules. Therefore, the lipase-holding capacity of PVP/PLMA-HGFMs decreased rapidly when the number of folds was increased, whereas the swelling ratio first increased and then decreased.

As shown in Figure 5e, with the change in UV irradiation time, the trend of lipase-holding capacity and swelling ratio of PVP/PLMA-HGFMs were similar to those of Figure 5d. The reasons for this were also similar. Conversely, the cause of pore reduction in this set of experiments was the increase in gel fraction. The amount of gel fraction was regulated by UV irradiation time: a longer irradiation time resulted in more adequate gelation of PVP-FMs and PLMA and a greater gel fraction.

### 2.5. Catalytic Properties of Lipases in PVP/PLMA-HGFMs

The relative specific activity of lipases in PVP/PLMA-HGFMs showed a trend of first increasing and then decreasing with the swelling of LMA solution and UV irradiation time increase (Figure 6a). This meant the relative specific activity was maximum only when these two variables were at the right values, respectively. The amount of PLMA in PVP/PLMA-HGFMs was swayed by the changes in swelling of LMA solution and UV irradiation time, but insufficient construction of the oil–water interfaces would be led by the ratio of the components of PVP-GFs and PLMA, which was either too large or too small. So, only when the ratio of the two reached a suitable value was it most advantageous for lipase catalysis.

Unlike Figure 6a, the relative specific activity of lipases decreased with increasing concentration and volume of lipase solution (Figure 6b). This was because the increase in lipase concentration led to an increase in the number of idle lipases in the aqueous phase at the same oil–water interfaces, and the increase in the volume of the lipase solution increased the aqueous phase and disrupted the oil–water interface equilibrium. All these factors hindered the increase in the relative specific activity of lipases.

The relative specific activity of lipases increased in both the experimental and control groups with increasing substrate concentrations (Figure 6c). The difference was that the rate of rise in the control group leveled off while the experimental group kept rising at a similar rate. This was also clearly shown by the folded lines in the graph. This meant the catalytic advantage of PVP/PLMA-HGFMs was more pronounced as the substrate concentration increased. This was caused by the different interface types in the control and experimental groups. Compared to the macroscopic “oil-up/water-down” interface in the control, the microscopic oil–water interface, which was smaller, more abundant, and had a more specific surface area in the experimental group, can simultaneously allow more substrates to participate in the reaction when the substrate concentration increases.

Figure 6d showed that the relative specific activity of lipases in both the experimental and control groups increased with the increase in folding times of PVP-FMs. The ratio of the two, however, was optimal (2.3) at folding times of three. This suggested the catalytic advantage of PVP/PLMA-HGFMs over the control was most pronounced at this point. For the experimental group, the increase in folding times of PVP-FMs led to a decrease in the pore size of PVP/PLMA-HGFMs and an increase in the mass transfer resistance, which, on the one hand, was unfavorable for the dispersion of substrate and lipases in the inner space, yet, on the other hand, was conducive to the formation of more catalytic interfaces between organic phase and aqueous phase. Thus, the changes brought by folding times were contradictory, and the optimal catalytic performance of PVP/PLMA-HGFMs was the combined result of this contradictory interaction.

### 2.6. Application Characteristics of PVP/PLMA-HGFMs

**Figure 7:**
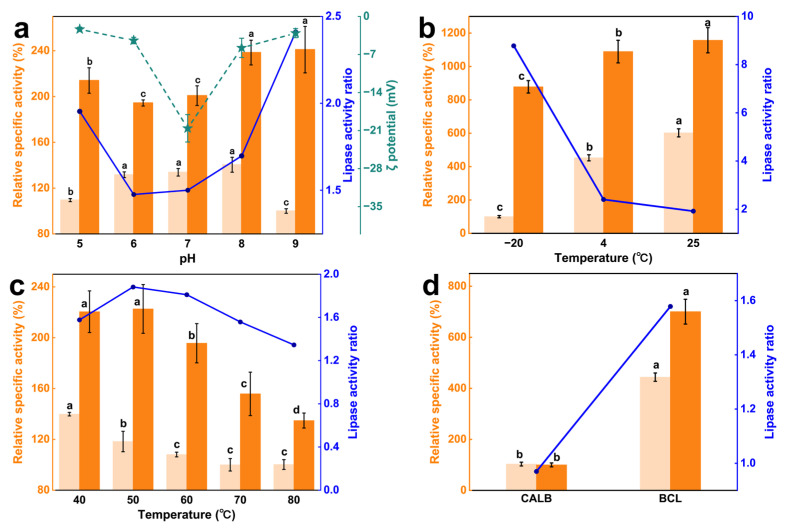
Application characteristics of PVP/PLMA-HGFMs. (**a**) Zeta potential of PVP/PLMA-HGFMs (green stars) and performance curve of lipase application of PVP/PLMA-HGFMs (blue dots) at different pH. (**b**) and (**c**) Curves of catalytic activity of lipase in PVP/PLMA-HGFMs at different temperatures. (**d**) Comparison of lipase catalytic activity of CALB and BCL. (**a**–**d**) The light yellow and orange in the bar graphs are for the control (“oil-up/water-down” system) and the experimental group (PVP/PLMA-HGFMs), respectively. Different letters in the same group indicate significant differences (*p* < 0.05).

It could be seen that the catalytic advantage of PVP/PLMA-HGFMs first decreased and then increased with increasing pH, and the catalytic advantage was lowest at neutral pH (Figure 7a). This indicated lipases had a certain acid-base tolerance due to PVP/PLMA-HGFMs. This was caused by the dipole moment of PVP/PLMA-HGFMs and lipases.

The dipole moment of PVP/PLMA-HGFMs was derived from the C=O of the side chains of PVP-GFs, and the dipole moment of lipases was derived from the polar and hydrophilic amino acids of helices α6 and α7 in the “lid” structure [33]. C=O with polarity was able to trigger charge distribution on solid-liquid surfaces [34,35]. Therefore, in a neutral environment, the catalytic system had a high (absolute) zeta potential (−20.58 mV). At this time, part of the C=O interacted with water molecules to form hydrogen bonds [36], while the other part of the C=O interacted with lipases through van der Waals forces. The space of lipase activity was restricted, resulting in fewer lipases participating in the catalytic process at the oil–water interfaces and a lower relative specific activity. When in an acidic or alkaline environment, C=O was more likely to interact with the large amounts of free H (+) or OH (−) in the environment and release lipases. This led to the zeta potential of the catalytic system tending to zero on the one hand, and on the other hand, it indirectly allowed more lipases to reach the oil–water interfaces, increasing the relative specific activity of lipases.

Figure 7b,c showed the relative specific activity of lipases in the experimental group was higher than that of the control in both low- and high-temperature environments. At −20 °C, the lipase activity ratio of the experimental group was 8.70 times higher than that of the control. Although low temperatures reduced the relative specific activity of lipases, the difference was that it declined more rapidly in the control group compared to the experimental group. This was due to the fact that the aqueous phase of the control froze at −20 °C, which made the mass transfer process of the catalytic reaction almost impossible. In contrast, the freezing point of PVP/PLMA-HGFMs was greatly reduced by the presence of PLMA organogel. Gao et al. [37] showed that heterogeneous network hybridized gels remain tough without brittleness at −80 °C. Thus, lipases are tolerant of low-temperature environments from PVP/PLMA-HGFMs.

Like low temperatures, high temperatures also inactivate lipases, but unlike the former, high temperatures destroy the spatial structure of lipases and made the inactivation of lipases irreversible. The relative specific activity of lipases in the control started to decrease at 40 °C, whereas in the experimental group, it was at 50 °C. This meant that the PVP/PLMA-HGFMs helped the lipase to have a little bit of resistance to high temperatures.

Overall, PVP/PLMA-HGFMs had catalytic advantages at both high and low temperatures, and this advantage was more pronounced at low temperatures.

In order to show that PVP/PLMA-HGFMs had a higher application value and catalytic advantage over lipases with a “lid” structure, a lipase without a “lid” structure, lipase B from Candida antarctica (CALB), was selected for study, and the relative specific activity of lipases was determined in the “oil-up/water-down” system (the control) and PVP/PLMA-HGFMs, respectively (the variable was the lipase type only). As shown in Figure 7d, the lipase activity ratio of BCL (1.58) with a “lid” structure was higher than that of CALB (0.97). This indicated that the lipase with a “lid” structure was one of the necessary conditions for the catalytic advantages of PVP/PLMA-HGFMs. This also indicated that the catalytic advantage of PVP/PLMA-HGFMs came from the optimization of the catalytic interfaces.

## 3. Conclusions

Starting from the catalytic interfaces of lipases, we constructed a novel lipase catalytic carrier, PVP/PLMA-HGFMs, by innovatively hybridizing hydrophilic PVP-FMs and lipophilic LMA in order to improve the catalytic efficiency of lipases. PVP/PLMA-HGFMs were able to create a large number of microscopic oil–water interfaces internally due to lots of pores, the interlaced network structure, and the ability to carry both water and oil. The catalytic efficiency of lipases was able to be increased 0.21–7.70 times under different experimental conditions. Furthermore, it was shown that lipases in PVP/PLMA-HGFMs exhibited tolerance to low temperatures (−20 °C) and extreme pH. 

Improving the reusability of lipase is one of the next focuses of this work (Figure S3). This implies that a new approach needs to take into consideration the immobilization strategy of lipase while inheriting the “interface catalysis” concept of this research. In conclusion, this work provided a new research idea for improving lipase activity and demonstrated the feasibility of hybrid gel-fiber membranes as carriers for lipase catalysis.

## 4. Materials and Methods

### 4.1. Materials

PVP (99%) was purchased from Boai NKY Pharmaceuticals Ltd. (Jiaozuo, China). LMA (96%) was purchased from Shanghai Bepharm Science & Technology Co., Ltd. (Shanghai, China). BCL (Amano Lipase PS) was purchased from Sigma-Aldrich (Hiroshima, Japan). *p*-Nitrophenyl palmitate (*p*-NPP) and *p*-nitrophenol (*p*-NP) were provided by Sigma-Aldrich (St. Louis, MO, USA). Ethylene dimethacrylate (EGDMA) and 2,2-diethoxyacetophenone (DEAP) were purchased from Shanghai Yuanye Bio-Technology Co., Ltd. (Shanghai, China). The phosphate buffer solution (PBS, pH = 7, 0.1 mol/L, ρ_PBS_ = 1016 mg/mL) used in the experiment was configured from disodium hydrogen phosphate and potassium dihydrogen phosphate. All other chemical reagents used in this study were of analytical grade and deionized water was used.

### 4.2. Preparation of PVP-FMs

Ten percent PVP (*w*/*w*) powder was weighed and dissolved in anhydrous ethanol, and the PVP electrospinning solution was obtained after magnetic stirring for 24 h at room temperature. To obtain the PVP-FMs, the PVP electrospinning solution was loaded into a 5 mL syringe with a separate propulsion pump at an ambient humidity of 30 ± 3%, a temperature of 25 ± 3 °C, a positive voltage of 12 kV, a negative voltage of −2 kV, a spinning needle distance of 12 cm from the receiver, an electrospinning rate of 0.05 mm/min and spun at a suitable angle for 8 h.

### 4.3. Preparation of PVP/PLMA-HGFMs

The PVP-FMs were folded several times and cut to the appropriate size, then immersed in a solution containing LMA (48 wt%), anhydrous ethanol, EGDMA (1 wt%) as a crosslinker, and DEAP (0.6 wt%) as a photoinitiator to fully swell. The saturated, solubilized PVP-FMs were subsequently subjected to high-pressure UV irradiation for 15 min. This process induced the formation of PVP-GFs and PLMA. The two were hybridized to become PVP/PLMA-HGFMs in the process.

### 4.4. Determination of Microscopic Morphology and Structure

The structural morphology of the samples was characterized microscopically using a scanning electron microscope (SEM, Zeiss G300, Oberkochen, BW, Germany). Gold-plating time is 1.5 min. The acceleration voltage is 1.5 kV.

### 4.5. Characterization of Microscopic Oil–Water Distribution

Confocal laser scanning microscopy (CLSM, Olympus FV3000, Tokyo, Japan) was used to observe the distribution of samples. For observation, PVP-GFMs and PLMA need to be stained with Nile blue (excitation wavelength of 640 nm) and Nile red (excitation wavelength of 488 nm) dyes, respectively.

### 4.6. Analysis of Fourier Transform Infrared Spectroscopy (FTIR)

The chemical groups of the samples were characterized by Fourier transform infrared spectroscopy (FTIR, Nicolet IS10, Thermo Nicolet Corporation, Madison, WI, USA). The scanning wavelength range is 400–4000 cm^−1^ with a resolution of 4 cm^−1^. Raw data were obtained for each sample using OMNIC32 (version number: 9.2.0.41) image processing software.

### 4.7. Test of Contact Angle (CA)

Water contact angle (WCA) and oil contact angle (OCA) were measured at room temperature using an optical contact angle meter (OCA, Biolin Theta Flex, Gothenburg, Sweden). The samples were cut into 1.2-cm-diameter discs and dried to ensure that there was no free water within the samples. Deionized water and dodecane were used as water and oil-wetting solutions, respectively. The volume of the droplet was controlled to be 1 mL. The results were recorded at the instant of contact between the droplet and the sample.

### 4.8. Zeta Potential

The PVP electrospinning spinning solution and LMA precursor solution were mixed in a ratio of 15.25:1 (*v*/*v*) and then diluted with PBS and its pH adjusted to 5, 6, 7, 8, and 9. Zeta potential determination was performed using a nanoparticle size and potential analyzer (Omec, NS-90Z Plus, Zhuhai, China).

### 4.9. Swelling Properties

The PVP/PLMA-HGFMs were cut into 1.2-cm-diameter discs and allowed to dissolve in PBS. The water swelling ratio and oil swelling ratio of the samples after a period of time were calculated according to Equation (1):

(1) $$\mathrm{Swelling}\;\mathrm{Ratio}\;(\%)=\frac{M_{T}{-M}_{0}}{M_{0}}\text{×}100\%$$

where M_T_ is the mass (g) of the sample after a period of swelling of time T, and M_0_ is the mass (g) of the sample before swelling.

Lipases were solubilized using PBS, and dried PVP/PLMA-HGFMs were placed in the lipase solution to make it swell. The ability (mg/g) of the sample to hold lipases after a certain time was calculated by Equation (2):

(2) $$\mathrm{Holding}\;\mathrm{Capacity}\;(\mathrm{mg}/g)=\frac{M_{1}-C_{T}\frac{M_{\mathrm{so}}-(M_{T}-M_{\mathrm{sa}})}{\rho_{\mathrm{so}}}}{M_{\mathrm{sa}}}$$

where M_1_ and M_so_ are the mass (mg) of lipases dissolved in PBS initially and the mass (g) of this lipase solution, respectively. M_sa_ is the original mass (g) of PVP/PLMA-HGFMs. PVP/PLMA-HGFMs were placed in the above lipase solution, and after a period of time T, the sample was removed and the lipase solution adhering to its surface was dried. At this point, the mass (g) of lipase solution absorbed by PVP/PLMA-HGFMs is expressed as M_T_, and C_T_ represents the concentration (mg/mL) of lipases in the remaining lipase solution measured by the BCA kit. Since the very small amount of lipases dissolved in PBS has a negligible effect on the density of PBS, ρ_so_ ≈ ρ_PBS_ = 1016 mg/mL.

### 4.10. Construction of Lipase Catalytic System and Lipases Activity

A certain volume and concentration of lipase solution was added dropwise to the surface of the PVP/PLMA-HGFMs and placed in 2 mL of *p*-NPP substrate solution (dodecane as solvent) after the lipase solution was fully absorbed. Lipases were catalyzed for a period of time, and 2 mL of anhydrous ethanol was added to terminate the reaction subsequently. The product (*p*-NP) was extracted using PBS, and its absorbance was measured at 410 nm using a microplate reader (Tecan Infinite M200 Pro, Tecan, Männedorf, Switzerland). The concentration of the product could be obtained according to Lambert-Beer law (Figure S1), which was then converted by the elution ratio (Figure S2) to obtain the actual concentration of the product. A unit of enzyme activity (U) was defined as the amount of enzyme required to produce 1 μmol of product in 1 min. Specific activity (U/mg), relative specific activity (%), and activity ratio were obtained from Equations (3), (4) and (5), respectively:

(3) $$\mathrm{Specific}\;\mathrm{Activity}\;\left(U/\mathrm{mg}\right)=\frac{U}{M_{\mathrm{lipase}}}$$

(4) $$\mathrm{Relative}\;\mathrm{Specific}\;\mathrm{Activity}\;\left(\%\right)=\frac{\mathrm{specific}\;\mathrm{activity}}{{\mathrm{specific}\;\mathrm{activity}}_{\min}}\text{×}100\%$$

(5) $$\mathrm{Lipase}\;\mathrm{Activity}\;\mathrm{Ratio}=\frac{U_{\mathrm{sample}}}{U_{\mathrm{control}}}$$

Equation (3) represents the enzyme activity (U) of 1 mg of lipases (M_lipase_, mg). Relative specific activity is defined as the ratio of the specific activity of one subject to the minimum value of the specific activity (specific activity_min_) of all subjects in a given one-way experiment (Equation (4)). Equation (5) represents the ratio of the enzyme activity of a particular experimental group (U_sample_) to its corresponding control group enzyme activity (U_control_). For all controls during the experiments (macroscopic “oil-up/water-down” system with *p*-NPP-containing dodecane as the organic phase and lipase-containing PBS as the aqueous phase), all conditions (cross-sectional area of the catalytic system, volume and concentration of lipase solution, etc.) were the same as in the corresponding experimental group, except for the difference in the type of the interface.

### 4.11. Statistical Analysis

Each experiment was repeated at least three times. Final results and errors were presented as mean and standard deviation, respectively. Origin 2024 was used for drawing and SPSS 26 was used for significance analysis (0.05) of the data.

## Data Availability

The data presented in this study are openly available in article.

## Supplementary Materials

The following supporting information can be downloaded at: https://www.mdpi.com/article/10.3390/gels10010074/s1, Figure S1: Standard curve of *p*-NP; Figure S2: Curve of elution ratio; Figure S3: Reusability of PVP/PLMA-HGFMs.
