# Supplementary material for: Novel Hybrid Gel–Fiber Membranes as Carriers for Lipase Catalysis Based on Electrospinning and Gelation Technology
Source: Gels. 2024 Jan 18;10(1):74. doi: 10.3390/gels10010074 (PMC10815851; doi:10.3390/gels10010074)
Supplement: Supplementary file 1 [file gels-10-00074-s001.zip › gels-2804671-supplementary.pdf]

gels

MDPI

Supplementary Materials

Novel Hybrid Gel–Fiber Membranes as Carriers for Lipase Catalysis Based on Electrospinning and Gelation Technology

Shumiao Lin [1], Qianqian Zhang [1], Ziheng Wang [2] and Jinlong Li [3,*]

[1] Beijing Engineering and Technology Research Center of Food Additives, Beijing Technology and Business University, Beijing 100048, China; linshumiao999@163.com (S.L.); 18852862233@163.com (Q.Z.)
[2] Key Laboratory of Green Manufacturing and Biosynthesis of Food Bioactive Substances, China General Chamber of Commerce, Beijing 100048, China; henghengheng2021@163.com
[3] School of Food and Health, Beijing Technology and Business University, Beijing 100048, China
* Correspondence: lijinlong@btbu.edu.cn; Tel.: +86-18500685896

1. Supplementary Materials

Nile red was purchased from Shanghai Macklin Biochemical Co., Ltd. (Shanghai, China). BCA kit was purchased from Beijing Solarbio Science & Technology Co., Ltd. (Beijing, China). Ethanol (100%), disodium hydrogen phosphate (≥99%) and potassium dihydrogen phosphate (≥99.5%) came from Fuchen (Tianjin) Chemical Reagent Co., Ltd. (Tianjin, China). Nile Blue was purchased from Shanghai Yuanye Bio-Technology Co., Ltd. (Shanghai, China).

2. Supplementary Methods

2.1. Plotting of standard curve

Lipase enzyme activity was determined by the *p*-nitrophenol (*p*-NP) colourimetric method. The catalytic activity of lipase was measured using *p*-NP as the chromogenic product. The *p*-NP standard curve: A certain amount of *p*-NP standard was dissolved in PBS buffer to form a *p*-NP standard solution with a concentration of 4 mg/mL, and placed in a refrigerator at 4℃ to protect it from light. The above *p*-NP standard solutions were configured into *p*-NP solutions with concentrations of 0.002 mg/mL, 0.004 mg/mL, 0.005 mg/mL, 0.008 mg/mL, 0.01 mg/mL, and 0.05 mg/mL with PBS buffer, respectively. A total of 200 μL of each concentration gradient of *p*-NP solution was added dropwise to a 96-well plate and the absorbance values at 410 nm were determined by a microplate reader.

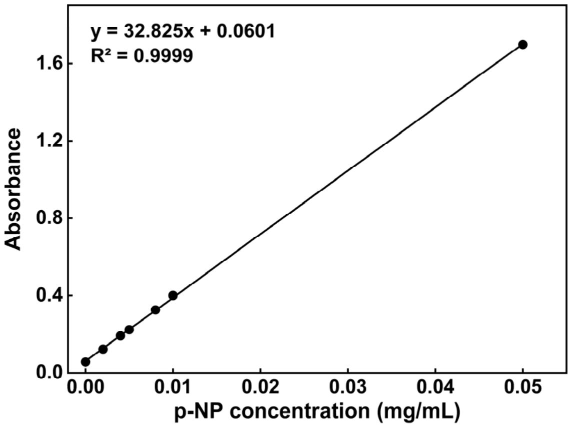

Figure S1. Standard curve of *p*-NP.

2.2. Determination of elution ratio

PVP/PLMA-HGFMs were placed in 2 mL of *p*-NP solution at a concentration of 4 mg/mL for several hours. When it was completely swelled remove and place it in a centrifuge tube. Next, 1 mL of PBS buffer (pH=7) was added to the centrifuge tube and stood for 5 minutes before removing PVP/PLMA-HGFMs and placing it in another centrifuge tube and repeating the above. The elution ratio of PVP/PLMA-HGFMs were determined at 410 nm using a microplate reader with different number of elution. The elution ratio was 90.06% after seven cycles.

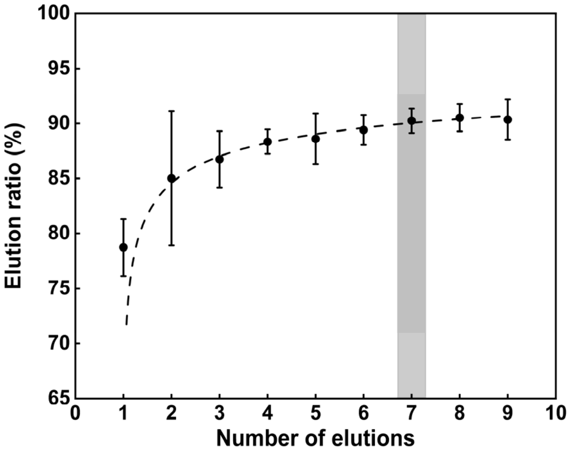

Figure S2. Curve of elution ratio.

2.3. Measurement of reusability

The PVP/PLMA-HGFMs lipase carrier was cycled several times at room temperature and pH = 7 to test its reusability. For a given cycle, the PVP/PLMA-HGFMs carrying a certain concentration and volume of lipase solution was placed in a certain concentration of 2 mL of substrate solution (dodecane as solvent) and reacted for a period of time. At the end of the reaction, 1 mL PBS buffer was added to the reaction device to extract the product *p*-NP remaining in the PVP/PLMA-HGFMs. After a period of extraction, the PVP/PLMA-HGFMs was removed from the reaction device and 2 mL of anhydrous ethanol was added to the device to terminate the lipase catalytic reaction that may be present. The removed PVP/PLMA-HGFMs were placed in another reaction device, and 2 mL of the above substrate solution was added to initiate the next round of catalysis by lipase. The enzyme activity of the first catalytic reaction of the samples in the group was defined as 100% relative specific activity.

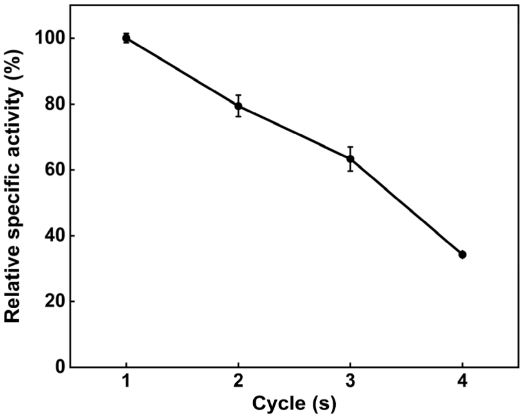

Figure S3. Reusability of PVP/PLMA-HGFMs.
